# Approximate Bayesian neural networks in genomic prediction

**DOI:** 10.1186/s12711-018-0439-1

**Published:** 2018-12-22

**Authors:** Patrik Waldmann

**Affiliations:** Department of Animal Breeding and Genetics, Swedish University of Agricultural Sciences (SLU), Box 7023, 750 07 Uppsala, Sweden

## Abstract

**Background:**

Genome-wide marker data are used both in phenotypic genome-wide association studies (GWAS) and genome-wide prediction (GWP). Typically, such studies include high-dimensional data with thousands to millions of single nucleotide polymorphisms (SNPs) recorded in hundreds to a few thousands individuals. Different machine-learning approaches have been used in GWAS and GWP effectively, but the use of neural networks (NN) and deep-learning is still scarce. This study presents a NN model for genomic SNP data.

**Results:**

We show, using both simulated and real pig data, that regularization is obtained using weight decay and dropout, and results in an approximate Bayesian (ABNN) model that can be used to obtain model averaged posterior predictions. The ABNN model is implemented in mxnet and shown to yield better prediction accuracy than genomic best linear unbiased prediction and Bayesian LASSO. The mean squared error was reduced by at least 6.5% in the simulated data and by at least 1% in the real data. Moreover, by comparing NN of different complexities, our results confirm that a shallow model with one layer, one neuron, one-hot encoding and a linear activation function performs better than more complex models.

**Conclusions:**

The ABNN model provides a computationally efficient approach with good prediction performance and in which the weight components can also provide information on the importance of the SNPs. Hence, ABNN is suitable for both GWP and GWAS.

## Background

Transformation of large quantities of data into valuable knowledge has become increasingly important in various fields of genomics and bioinformatics [1]. Machine-learning methods are flexible general-purpose approaches to automatically learn complex relationships and patterns from data, and they play a vital role in the analysis of big data [2–4]. The concept of genome-wide prediction (GWP) was introduced by Meuwissen et al. [5] and refers to the idea that regression coefficients of genomic markers, often single-nucleotide polymorphisms (SNPs), can be used to predict phenotypes of individuals. In order to identify markers that affect some phenotype of interest, state of the art genome-wide marker data comprise several thousands, sometimes millions of SNPs, scored in a number of individuals that is in the order of some hundreds to a few thousands [6]. There are plenty of examples of the successful use of machine-learning in genomic prediction, genome-wide association studies and in other forms of genomic sequence analysis [7, 8].

Among the most flexible methods in machine-learning are deep artificial neural networks, which have recently received large attention because of their outstanding prediction properties [9]. An artificial neural network (NN) connects the inputs (predictor variables) to an output (response variable), either directly or through one or several layers of interconnected computing units (neurons). The depth of an NN corresponds to the number of hidden layers and the width to the number of neurons in its layers. NN with larger numbers of hidden layers are called “deep networks”. Training of a NN is accomplished with mathematical optimization algorithms that iteratively perform forward and backward passes (epochs) in order to minimize some loss (error) function and learn the weights (regression coefficients) and biases (intercepts) of the inputs. In the forward pass, the linear or non-linear activation functions are applied to the current values of the weights of the links to get the output at each layer. The final result of a forward pass is new predicted outputs. The backward pass starts by calculating the derivatives of the error function between the predicted outputs and the real outputs. Then, the derivatives are propagated backwards updating the weights and computing new error terms for that layer. This process is repeated for each layer until the input layer is reached again [10]. The number of epochs and the learning rate determine the amount of training and need to be evaluated against validation or test data in order to avoid over-fitting.

Gradient descent is a popular algorithm that is used to perform mathematical optimization and is one of the most common ways to perform learning in neural networks. The gradient is computed layer-wise using the chain rule for reverse-mode differentiation [11]. The computed gradient indicates by what amount the errors would increase or decrease if the weights are increased by a small amount. Then, the weight vector is adjusted in the opposite direction to the gradient vector, i.e. the negative gradient vector specifies the direction of the steepest descent towards the minimum of the loss function. The gradient descent algorithm is an efficient alternative in NN with many connections, but easily leads to over-fitting. Two of the most important methods for regularization in NN are weight decay and dropout [12]. Weight decay is an old technique in which each weight decays towards zero at a rate that is proportional to its magnitude. Weight decay is closely related to ridge regression and can be interpreted as Bayesian Gaussian regularization [13]. Dropout is a recent innovation where a random proportion of the input weights are set to zero in each epoch [14]. Recently, it was shown that dropout can be interpreted as a Bayesian approximation to deep-learning with a near connection to Gaussian processes [15].

In genetics, there are some examples of the use of NN. Gianola et al. [16] introduced the feed-forward NN and showed how it could be interpreted in terms of standard regression models, and classical and molecular genetics. Moreover, they suggested Bayesian ridge regression based regularization to prevent overfitting in the NN and showed that this improved prediction accuracy of traits such as milk production in Jersey cows and yield of inbred lines of wheat compared to normal linear models. The same model improved prediction accuracy of body mass index in mice in an accompanying study [17]. Ehret et al. [18] replaced the computationally demanding Levenberg–Marquardt training algorithm in [16] with back-propagation. Glória et al. [19] showed how two approximate measures of variable contribution and importance could be used to assess marker effects in genomic NN. Deep-learning has also received attention in the related fields of bioinformatics and systems biology [20, 21].

The purpose of this study is to present a NN model for genomic SNP data that can be modified easily. We will also show that regularization is obtained using weight decay and dropout, and that it results in an approximate Bayesian model that can be used to obtain model-averaged posterior predictions. The predictive accuracy of the NN model is evaluated by using both simulated and real data, and we show that weight components can provide information on the importance of SNPs under some circumstances.

## Methods

### Neural network

The most basic model starts with a single hidden layer NN. Denote ${\textbf{W}}_{1}$ and ${\textbf{W}}_{2}$ the weight matrices that connect the input matrix ${\textbf{X}}$ of dimension $n \times p$ to the output ${\textbf{y}}$ of dimension $n \times 1$ through the hidden layer. The dimensions of ${\textbf{W}}_{1}$ and ${\textbf{W}}_{2}$ depend on the number of units in the hidden layer. Hence, for a hidden layer with $k$ units ${\textbf{W}}_{1}$ will be of dimension $p \times k$ and ${\textbf{W}}_{2}$ of dimension $k \times 1$. Associated with ${\textbf{W}}_{1}$ and ${\textbf{W}}_{2}$ is a bias vector ${\textbf{b}}$ of dimension $k$ and an activation function $\sigma ( \cdot )$ that performs element-wise linear or non-linear transformation of the inputs. A standard NN model now becomes:

1 $${\hat{\textbf{y}} = \sigma \left( {{\textbf{X}}{\textbf{W}}_{1} + {\textbf{b}}} \right){\textbf{W}}_{2}} .$$

A model with one hidden layer containing one unit will be of dimension $1 \times 1$ and thus, ${\textbf{W}}_{2}$ disappears. For regression purposes, the NN model is usually completed with the Euclidean squared loss function:

2 $${\mathcal{L}}\left( {{\mathbf{y}},{\hat{\mathbf{y}}}} \right) = \frac{ 1}{ 2n}\mathop \sum \limits_{i = 1}^{n} \left\| {y_{i} - \hat{y}_{i} } \right\|_{2}^{2} ,$$

where $|| \cdot ||_{2}^{2}$ denotes the square of the Euclidean norm. For classification, one can use the logistic or cross-entropy loss.

If $p$ is larger than $n$, training of the NN will lead to over-fitting and it is necessary to regularize the NN parameters ${\varvec{\uptheta}} = \left\{ {{\textbf{W}}_{1} ,{\textbf{W}}_{1} , {\textbf{b}}} \right\}$. Therefore, regularization terms are added during optimization. One common approach is to add the ${\ell}_{2}$ penalty [4] through weight decay parameters $\lambda_{i}$, which need to be tuned by cross-validation. The cost function to minimize is now:

3 $${\text{minimize}}\left\{ {J\left( {\varvec{\uptheta}} \right) = \frac{ 1}{ 2n}\mathop \sum \limits_{i = 1}^{n} \left\| {y_{i} - \hat{y}_{i} } \right\|_{2}^{2} + \lambda_{1} \left\| {{\mathbf{W}}_{1} } \right\|_{2}^{2} + \lambda_{2} \left\| {{\mathbf{W}}_{2} } \right\|_{2}^{2} + \lambda_{3} \left\| {\mathbf{b}} \right\|_{2}^{2} } \right\}.$$

The above single hidden layer NN with the Euclidean loss is identical to a basis function regression model. With $k = 1$ and $\sigma ( \cdot )$ chosen to be the linear identity function, the model is closely related to ridge regression. Extending this simple NN model to multiple layers is straightforward and results in a more expressive deep NN model, but it may result in over-fitting if the relationship between the input and output is linear and lacks structure. Hence, it is important to evaluate NN with different architectures and possibly also different activation functions.

### Activation functions and one-hot encoding

The most simple activation function is the linear identity function $\sigma \left( x \right) = x$, which has the derivative $\sigma '\left( x \right) = 1$ and range $\left( { - \infty ,\infty } \right)$, and therefore is well adapted for linear regression purposes. In genetic terms, this function will infer additive effects of bi-allelic loci if the SNPs are coded 0, 1 and 2. In order to model non-linear effects, there are several activation functions that can be used. The tanh function is $\sigma \left( x \right) = \left( {2/\left( {1 + e^{ - 2x} } \right)} \right) - 1$ with derivative $\sigma^{'\left( x \right)} = 1 - \sigma \left( x \right)^{2}$ and range $\left( { - 1, 1} \right)$. The rectifier function is defined as $\sigma \left( x \right) = { \hbox{max} }\left( {0, x} \right)$ and has several related functions, for example the rectified linear unit (ReLU). However, these non-linear functions may not be so useful for the first layer if the input contains integer coding (e.g. the 0, 1 and 2 of SNP genotypes) and the weights are expected to be in both the positive and negative domains.

An alternative way of inferring non-linearity of integer coded input variables is one-hot encoding [22]. In this approach, indicator variables are formed for each level of the input variable. From a genetic perspective, it means that each genotype will be coded by one $\left( {0, 1} \right)$ variable, and both additive and dominance effects can be modelled straightforwardly.

### Optimization algorithms

Gradient descent (GD) is a first order optimization algorithm where the cost function $J\left( {\varvec{\uptheta}} \right)$ is minimized by updating the parameters in the opposite direction of the gradient of the cost function $\nabla_{{\varvec{\uptheta}}} J\left( {\varvec{\uptheta}} \right)$. The learning rate $\eta$ determines the size of the steps towards the minimum. The original gradient descent uses all training data per epoch $t$:

4 $${\mathbf{g}}_{t} = \nabla_{{{\varvec{\uptheta}}_{t - 1} }} J\left( {{\varvec{\uptheta}}_{t - 1} } \right),\quad{\varvec{\uptheta}}_{t} = {\varvec{\uptheta}}_{t - 1} - \eta {\mathbf{g}}_{t} ,$$

and, thus, can be slow for large datasets and get stuck in local minima. In contrast, stochastic gradient descent (SGD) uses random single training samples that modifies the gradient to:

5 $${\mathbf{g}}_{t} = \nabla_{{{\varvec{\uptheta}}_{t - 1} }} J\left( {{\varvec{\uptheta}}_{t - 1} ;y_{i} ;{\mathbf{x}}_{i} } \right),$$

which makes it much faster per epoch, but also results in large fluctuations of the cost function. A compromise between GD and SGD is mini-batch GD (BSGD), where a batch of size $b$ is used for every epoch:

6 $${\mathbf{g}}_{t} = \nabla_{{{\varvec{\uptheta}}_{t - 1} }} J\left( {{\varvec{\uptheta}}_{t - 1} ;y_{i \ldots i + b} ;{\mathbf{x}}_{i \ldots i + b} } \right),$$

which reduces the variance between updates and makes computations efficient. The number $b$ needs to be chosen, usually between 50 and 250 depending on the number of observations in the training data.

Several suggestions on how to improve the convergence properties of BSGD [23] have been reported. Kingma and Ba [24] introduced the adaptive moment estimation (ADAM) algorithm that computes momentum components of the gradients:

7 $${\mathbf{m}}_{t} = \beta_{1} {\mathbf{m}}_{t - 1} + \left( {1 - \beta_{1} } \right){\mathbf{g}}_{t} ,\,{\mathbf{v}}_{t} = \beta_{2} {\mathbf{v}}_{t - 1} + \left( {1 - \beta_{2} } \right){\mathbf{g}}_{t}^{2} ,$$

where ${\mathbf{m}}_{t}$ is the moving average of the gradient and ${\mathbf{v}}_{t}$ is the moving average of the squared gradient. $\beta_{1}$ and $\beta_{2}$ are hyper-parameters that control the exponential decay of the moving averages. Furthermore, their bias-corrected estimates are calculated as:

8 $${\hat{\mathbf{m}}}_{t} = \frac{{{\mathbf{m}}_{t} }}{{1 - \beta_{1}^{t} }},\quad{\hat{\mathbf{v}}}_{t} = \frac{{{\mathbf{v}}_{t} }}{{1 - \beta_{2}^{t} }},$$

which leads to an update of the parameters with adaptive learning rate:

9 $${\varvec{\uptheta}}_{t} = {\varvec{\uptheta}}_{t - 1} - \eta \frac{{{\hat{\mathbf{m}}}_{t} }}{{\sqrt {{\hat{\mathbf{v}}}_{t} } + \epsilon}},$$

where $\epsilon$ controls the effective stepsize. Kingma and Ba [24] suggest the following default hyperparameters, $\beta_{1} = 0.9$, $\beta_{2} = 0.999$ and $\epsilon = 10^{ - 8}$. ADAM can be combined with weight decay, and then will perform ${\ell}_{2}$ regularization. This results in parameter update:

10 $${\varvec{\uptheta}}_{t} = \left( {1 - \frac{\eta \lambda }{b}} \right){\varvec{\uptheta}}_{t - 1} - \eta \frac{{{\hat{\mathbf{m}}}_{t} }}{{\sqrt {{\hat{\mathbf{v}}}_{t} } + \epsilon}}.$$

### Dropout and its Bayesian interpretation

Dropout is applied by sampling of binary vectors ${\textbf{z}}_{1}$ and ${\textbf{z}}_{2}$ in each epoch from two Bernoulli distributions, i.e. ${\textbf{z}}_{1} \sim {\text{Bernoulli}}\left( {p_{1} } \right)$ and ${\textbf{z}}_{2} \sim {\text{Bernoulli}}\left( {p_{2} } \right)$, and setting $1 - p_{1}$ of the inputs and $1 - p_{2}$ of the outputs to zero. This leads to an extension of Eq. (1) as follows:

11 $${\hat{\mathbf{y}}} = \sigma \left( {{\textbf{X}}\left( {{\textbf{z}}_{1} {\textbf{W}}_{1} } \right) + {\textbf{b}}} \right)\left( {{\textbf{z}}_{2} {\textbf{W}}_{2} } \right).$$

The dropped weights of ${\textbf{z}}_{1} {\textbf{W}}_{1}$ and ${\textbf{z}}_{2} {\textbf{W}}_{2}$ are usually scaled by $1/p_{1}$ and $1/p_{2}$, respectively, to maintain constant output magnitude. The surviving nodes have to stand in for those that are omitted, which forms another form of regularization that has been shown to be effective in preventing over-fitting [25]. Dropout can be interpreted in several ways [26, 27]. Gal and Ghahramani [15] showed that dropout is mathematically equivalent to a variational approximation of a Bayesian deep Gaussian process. The goal of Bayesian prediction is the posterior predictive distribution [28], which for the test input ${\mathbf{X}}^{\varvec{*}}$ is:

12 $$p\left( {\left. {{\hat{\mathbf{y}}}^{\varvec{*}} } \right|{\mathbf{X}}^{\varvec{*}} ,{\mathbf{X}},{\mathbf{y}}} \right) = \int {p\left( {\left. {{\hat{\mathbf{y}}}^{\varvec{*}} } \right|{\mathbf{X}}^{\varvec{*}} ,{\varvec{\upomega}}} \right)p\left( {\left. {\varvec{\upomega}} \right|{\mathbf{X}},{\mathbf{y}}} \right){\text{d}}{\varvec{\upomega}}} ,$$

where $p({\hat{\mathbf{y}}}^{\varvec{*}} |{\mathbf{X}}^{\varvec{*}} ,{\varvec{\upomega}})$ is the likelihood of the test output (response) and ${\varvec{\upomega}} = \left\{ {{\textbf{z}}_{1} {\textbf{W}}_{1} ,{\textbf{z}}_{2} {\textbf{W}}_{2} ,{\textbf{b}}} \right\}$. The posterior distribution of the training data $p\left( {\left. {\varvec{\upomega}} \right|{\mathbf{X}},{\mathbf{y}}} \right)$ is usually analytically intractable, but the variational distribution $q\left( {\varvec{\upomega}} \right)$ can be defined as:

$$\begin{aligned} & {\textbf{W}}_{i} = {\textbf{M}}_{i} \cdot {\text{diag}}\left( {\left[ {z_{i,j} } \right]_{j = 1}^{k} } \right), \\ & {z}_{i,j} \sim {\text{Bernoulli}}\left( {p_{i} } \right), \\ \end{aligned}$$

where $\left\{ {i = 1, 2} \right\}$, $\left\{ {j = 1, \ldots ,k_{i - 1} } \right\}$, ${\textbf{M}}_{i}$ is a random variational matrix and $p_{i}$ are the dropout probabilities. $p_{1}$ and $p_{2}$ were set to 0.5 for all NN configurations in this study.

Then, the idea behind variational inference is to minimize the Kullback–Leibler (KL) divergence between $q\left( {\varvec{\upomega}} \right)$ and $p\left( {\left. {\varvec{\upomega}} \right|{\mathbf{X}},{\mathbf{y}}} \right)$ through maximization of the log evidence lower bound:

13 $${\mathcal{L}}_{VI} = \int {q\left( {\varvec{\upomega}} \right){\text{log }}p\left( {\left. {\varvec{\upomega}} \right|{\mathbf{X}},{\mathbf{y}}} \right){\text{d}}{\varvec{\upomega}} - {\text{KL}}\left( {q\left( {\varvec{\upomega}} \right)p\left( {\varvec{\upomega}} \right)} \right)} ,$$

which results in the approximate predictive distribution:

14 $$q\left( {\left. {{\hat{\mathbf{y}}}^{\varvec{*}} } \right|{\mathbf{X}}^{\varvec{*}} } \right) = \int {p\left( {\left. {{\hat{\mathbf{y}}}^{\varvec{*}} } \right|{\mathbf{X}}^{\varvec{*}} ,{\varvec{\upomega}}} \right)q\left( {\varvec{\upomega}} \right){\text{d}}{\varvec{\upomega}}} .$$

Note that the loss function is equal to the negative log-likelihood, i.e. ${\mathcal{L}}\left( {{\mathbf{y}},{\hat{\mathbf{y}}}} \right) = - {\text{log }}p\left( {\left. {\mathbf{y}} \right|{\mathbf{X}},{\varvec{\upomega}}} \right)$, and that the dropout model can be interpreted as Bayesian ridge regression with a spike-and-slab *g*-prior [29]. Sampling from Eq. (13) is straightforward. Start by sampling $T$ sets of vectors $\left\{ {{z}_{i,j}^{t} } \right\}_{t = 1}^{T} \sim {\text{Bernoulli}}\left( {p_{i} } \right)$, combine with ${\textbf{W}}_{i}$ to obtain $\left\{ {{\mathbf{W}}_{i}^{t} } \right\}_{t = 1}^{T}$ and perform one gradient descent optimization per $t$ with some predefined values of the weight decay. ADAM will automatically adapt the learning rate. Iterate until $T$ to get predictions $\left\{ {{\hat{\mathbf{y}}}^{{\varvec{*}t}} } \right\}_{t = 1}^{T}$ and calculate ${\text{MSE}}_{t}$ for each iteration:

15 $${\text{MSE}}_{t} = \frac{1}{ntest}\mathop \sum \limits_{1}^{ntest} \left( {{\hat{\mathbf{y}}}^{{\varvec{*}t}} - {\mathbf{y}}_{test} } \right)^{2} .$$

Plot the ${\text{MSE}}_{t}$ against the iteration number to determine at which iteration $t_{s}$ the chain has converged. The first moments (expectations) of the parameters ${\varvec{\upomega}}$ are approximated as:

16 $${\mathbb{E}}\left[ {\varvec{\upomega}} \right] \approx \frac{1}{{T - t_{s} }}\mathop \sum \limits_{{t = t_{s} + 1}}^{T} {\varvec{\upomega}}^{t} ,$$

whereas the predictive variances for the parameters are:

17 $${\text{VAR}}\left[ {\varvec{\upomega}} \right] \approx \frac{1}{{T - t_{s} }}\mathop \sum \limits_{{t = t_{s} + 1}}^{T} \left( {{\varvec{\upomega}}^{t} - {\mathbb{E}}\left[ {{\varvec{\upomega}}^{t} } \right]} \right)^{2} .$$

Moreover, the model’s averaged MSE can be calculated based on the MSE from each $t$ of the stationary part of the chain yielding:

18 $${\text{MSE}}_{{\mathcal{M}}} = \frac{1}{{T - t_{s} }}\mathop \sum \limits_{{t = t_{s} + 1}}^{T} \frac{1}{ntest}\mathop \sum \limits_{1}^{ntest} \left( {{\hat{\mathbf{y}}}^{{\varvec{*}t}} - {\mathbf{y}}_{test} } \right)^{2} .$$

### Data

#### Simulated data with dominance

The original data was produced for the QTLMAS2010 workshop and intended to mimic a real breeding livestock population [30]. The number of individuals is 3226, and these are structured in a pedigree with five generations. The pedigree is founded by 20 individuals (5 males and 15 females), and it was created assuming that each female mates once and gives birth to approximately 30 progeny. Five 100 Mbp long autosomal chromosomes were simulated. A neutral coalescent model was used to simulate the SNP data. The algorithm created 10,031 markers, including 263 monomorphic and 9768 biallelic SNPs. The mean LD ($r^{2}$) between adjacent SNPs with a minor allele frequency (MAF) higher than 0.05 is estimated at 0.100 (SD = 0.152).

The continuous quantitative trait was created from 37 quantitative trait loci (QTL), including nine controlled genes and 28 random genes. The QTL were modelled as additive effects, apart from two pairs of additive epistatic QTL and three paternal imprinting QTL. The controlled genes were selected based on their high polymorphism as well as their high linkage disequilibrium (LD) with markers. The additive effects of all controlled QTL were equal to +3 (i.e. half the difference between the means of the homozygotes). The random genes were selected from the simulated SNPs and then their effects were sampled from a truncated normal distribution, $N\left( {0,10} \right)$, and accepted if the absolute value of the additive effect was less than 2. The resulting additive effects of the random genes varied between − 1.98 and 1.93. The two epistatic pairs of QTL are on chromosomes 1 and 2, respectively, and determined by four controlled additive QTL with an additional epistatic effect of 4 for the lower homozygote pairs. Each simulated QTL was surrounded by 19 to 47 polymorphic SNPs (MAF > 0.05) positioned within a 1-Mb distance from the QTL. A total of 364 SNPs were in moderate to high LD with the QTL ($r^{2} > 0.1$).

Furthermore, one dominance locus was positioned at SNP 9212 on chromosome 5 by giving the heterozygote (1) an effect of 5, and the upper homozygote (2) a value of 5.01 (for numerical reasons). One over-dominance locus was produced at SNP 9404 by assigning the heterozygote an effect of 5, the lower homozygote (0) an effect of − 0.01, and the upper homozygote (2) an effect of 0.01. Finally, one under-dominance loci was created at SNP 9602 by assigning a value of − 5 to the heterozygote, and giving the lower homozygote (0) an effect of − 0.01 and the upper homozygote (2) an effect of 0.01. The values of the genotypes of these new SNPs were added to the original **y**-values. SNPs with a MAF lower than 0.01 were removed resulting in a final sample of 9723 SNPs.

#### Real data

Cleveland et al. [31] published a pig dataset comprising 3534 individuals with high-density genotypes, phenotypes, and estimated breeding values for five anonymous traits. Genotypes were obtained with the PorcineSNP60 chip, and after quality control, 52,842 SNPs remained. Missing genotypes were imputed using a probability score. SNPs with both known and unknown positions were included and imputed. The map order was randomized and the SNP identities were recoded. The number of SNPs was further reduced in this study by a more stringent MAF (< 0.01), which produced a final number of 50,276 SNPs.

Most of the genotyped animals were measured for all five purebred traits (phenotypes in a single nucleus line). Heritabilities ranged from 0.07 to 0.62. In this study, trait 3 with a heritability of 0.38 was used. The phenotypic data points were adjusted for environmental factors and rescaled by correcting for the overall mean. Individuals with missing phenotype data were removed which, at the end, resulted in 3141 observations.

### Implementation

All NN models were implemented in the Python version of MXNet [32] using the ADAM optimizer with default settings. The Python code is available at https://github.com/patwa67/ABNN. Predictions were also obtained for Bayesian LASSO (BLASSO) and genomic best linear unbiased prediction (GBLUP) (using default settings in the R-package BGLR; [33]). For the simulated QTLMAS2010 datasets, individuals in generations 1 to 4 (2326 individuals) were used as training data and in generation 5 (900 individuals) as test data. Individuals in the real pig data were divided into different cross-validation (CV) sets with test datasets of sizes between 627 and 631. The MSE was averaged over these CV sets.

## Results

### Simulated data

The Monte Carlo Markov chains of the GBLUP and BLASSO analyses were run for 60,000 iterations, and a burn-in of 10,000 and thinning of 10 resulted in a final sample of 5000 iterations. The resulting testing set MSE was 88.42 and 89.22 for the GBLUP and BLASSO, respectively (Table 1). Initially, two NN were tested in which the first one was designed to have one hidden layer and one node, and the second to have two hidden layers with two and one node, respectively. The weight decay were varied between 1.0 and 1.5 for both NN. The ABNN analyses were run for 6000 iterations and the first 1000 were considered as burn-in. Overall, the model averaged test MSE and its standard deviation were smaller for the first NN than for the second NN (Table 1). This NN also produced the smallest test MSE with an estimate of 82.69 for a weight decay of 1.4.

**Table 1:** Test set MSE for GBLUP, BLASSO and ABNN evaluated on the simulated QTLMAS2010 data

GBLUP	88.42					
BLASSO	89.22					
ABNN	Weight decay $\lambda_{1}$					
	1.0	1.1	1.2	1.3	1.4	1.5
# units $k$ = 1						
${\text{MSE}}_{{\mathcal{M}}}$ (SD)	83.64 (0.272)	83.26 (0.216)	83.27 (0.243)	83.50 (0.256)	*82.69* (0.218)	83.51 (0.256)
# units $k$ = 2.1						
${\text{MSE}}_{{\mathcal{M}}}$ (SD)	88.40 (0.940)	87.94 (0.733)	89.31 (1.167)	88.12 (0.840)	87.42 (0.714)	88.01 (0.727)

Two architectures were evaluated for the ABNN were $k$ refers to the number of units per hidden layer. ${\text{MSE}}_{{\mathcal{M}}}$ is the model-averaged MSE and SD is the standard deviation over iterations excluding burn-in. The best model MSE is indicated in italic characters

**Fig. 1:**
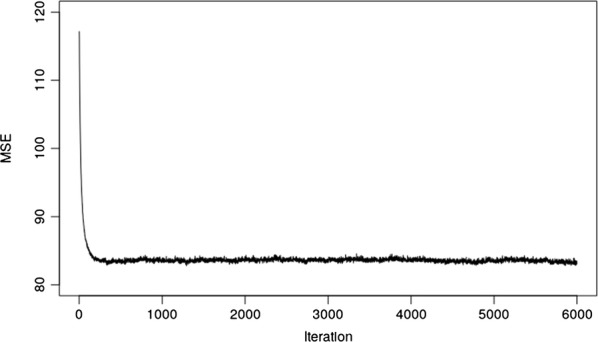
Trace plot of test MSE for the best model (one-neuron, one-layer and linear activation function) on the simulated QTLMAS2010 data

The MSE quickly converged to a stationary phase for this NN (Fig. 1). One larger NN with three layers (3, 2 and 1 nodes, respectively) was run only for a weight decay of 1.4. The resulting test MSE was equal to 93.55 with a standard deviation (sd) of 1.424. In addition, the best model was evaluated with tanh and relu activation functions. The resulting test MSE were equal to 85.68 (sd = 0.166) and 83.72 (sd = 0.248), respectively.

In order to investigate the effect of possible outliers in the test predictions, we also calculated the model averaged mean absolute error (MAE) for the most important models. MAE was equal to 7.640 and 7.635 for the BLASSO and GBLUP, respectively. The smallest MAE was 7.407 for the small ABNN model with a weight decay of 1.5. The MAE for the larger ABNN model was 7.675, which indicates overall the same pattern as for MSE.

**Fig. 2:**
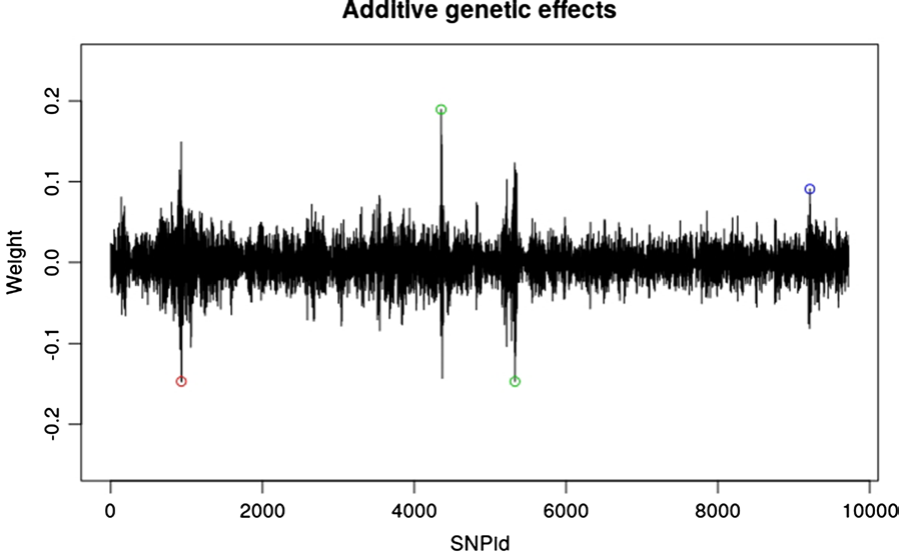
Mode-averaged weight plots for the additive genetic effects for the best model (one-neuron, one-layer and linear activation function) on the simulated QTLMAS2010 data. The two major additive loci are SNP 4354 and 5327 (red circles) and the additive part of the epistatic effect at SNP 931 is indicated by a red circle) and the additive part of the dominance locus at SNP 9212 by a blue circle

**Fig. 3:**
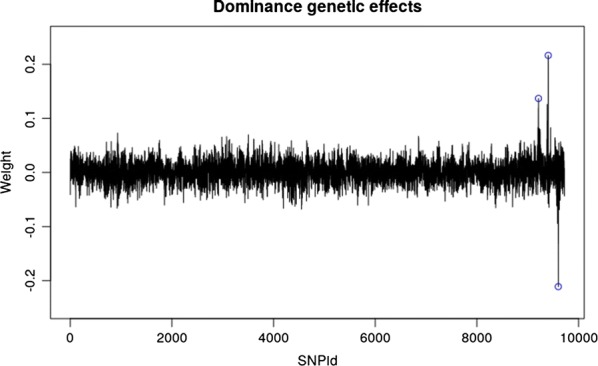
Model-averaged weight plots for the dominance genetic effects for the best model (one-neuron, one-layer and linear activation function) on the simulated QTLMAS2010 data. The dominance, over-dominance and under-dominance loci were identified at SNPs 9212, 9404 and 9602, respectively (blue circles)

The model’s averaged weights ${\mathbb{E}}\left[ {{\mathbf{W}}_{1} } \right]$ were calculated for the best model. The additive genetic effects were then obtained from the two homozygotes as $a = - {\mathbb{E}}\left[ {{\mathbf{W}}_{{1,{\text{Hom}}0}} } \right]$ + ${\mathbb{E}}\left[ {{\mathbf{W}}_{{1,{\text{Hom}}2}} } \right]$ and are plotted in Fig. 2. It can be seen that the ABNN detects the two major additive loci on chromosome 3 (SNPs 4354 and 5327) and the additive part of the epistatic effect on chromosome 1 (SNP 931) as well as the additive part of the dominance locus (SNP 9212). Then, the dominance genetic effects were obtained from the heterozygote as $d = {\mathbb{E}}\left[ {{\mathbf{W}}_{{1,{\text{Het}}1}} } \right]$ and are plotted in Fig. 3. It is clear that dominance, over-dominance and under-dominance loci were identified at SNP positions 9212, 9404 and 9602, respectively.

### Real data

The analyses of the real data were run with the same number of iterations as for the simulated data. For the GBLUP and BLASSO, the MSE estimates were equal to 0.8759 and 0.8741, respectively (Table 2). In the ABNN analyses, weight decays were optimized for values between 21 and 25 based on the two smallest NN. Again, the model averaged MSE and its standard deviation were overall smaller for the first NN than for the second NN, with MSE estimates of 0.8653 and 0.9221, respectively, for a weight decay of 23 (Table 2). For the third NN, the test MSE was equal to 0.9236 (sd = 0.00447). The analyses of the best model with tanh and relu activation functions resulted in test MSE of 0.894 (sd = 0.000383) and 0.867 (sd = 0.000781), respectively.

**Table 2:** Test set MSE for GBLUP, BLASSO and ABNN evaluated on the real Cleveland pig dataset

GBLUP	0.8759				
BLASSO	0.8741				
ABNN	Weight decay $\lambda_{1}$				
	21	22	23	24	25
# units $k$ = 1					
${\text{MSE}}_{{\mathcal{M}}}$ (SD)	0.8688 (0.000796)	0.8675 (0.000790)	*0.8653* (0.000722)	0.8676 (0.000741)	0.8687 (0.000728)
# units $k$ = 2.1					
${\text{MSE}}_{{\mathcal{M}}}$ (SD)	0.9230 (0.00432)	0.9233 (0.00440)	0.9221 (0.00399)	0.9233 (0.00430)	0.9235 (0.00439)

Two architectures were evaluated for the ABNN were $k$ refers the number of units per hidden layer. ${\text{MSE}}_{{\mathcal{M}}}$ is the model-averaged MSE and SD is the standard deviation over iterations excluding burn-in. The best model MSE is indicated in italic characters

MAE of 0.6863 and 0.6865 were obtained for the BLASSO and GBLUP models, respectively. The smallest MAE was 0.6811 for the small ABNN model with a weight decay set to 22. The second ABNN model yielded a MAE of 0.7153 for a weight decay of 22. Hence, the pattern of MAE is very similar to the MSE pattern for both datasets.

**Fig. 4:**
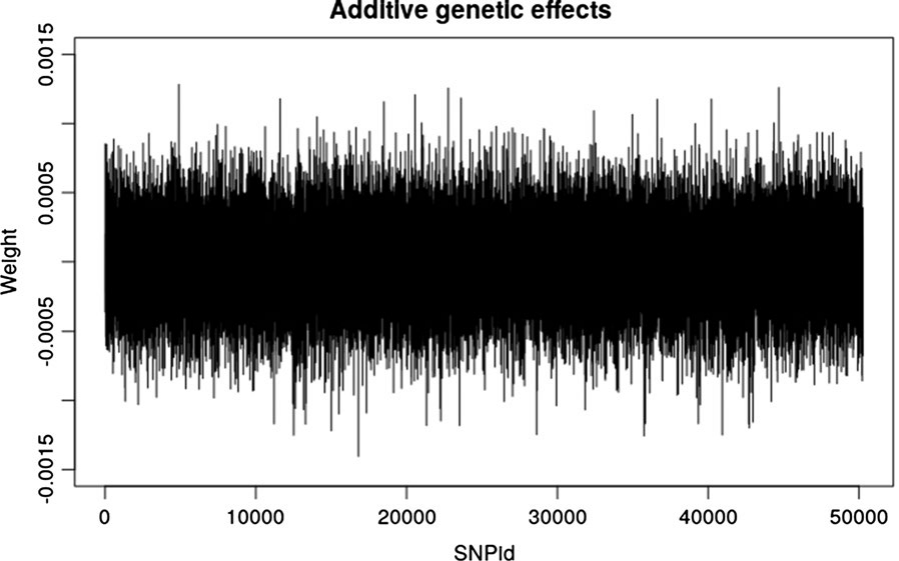
Model-averaged weight plots for the additive genetic effects for the best model (one-neuron, one-layer and linear activation function) on the Cleveland pig dataset

**Fig. 5:**
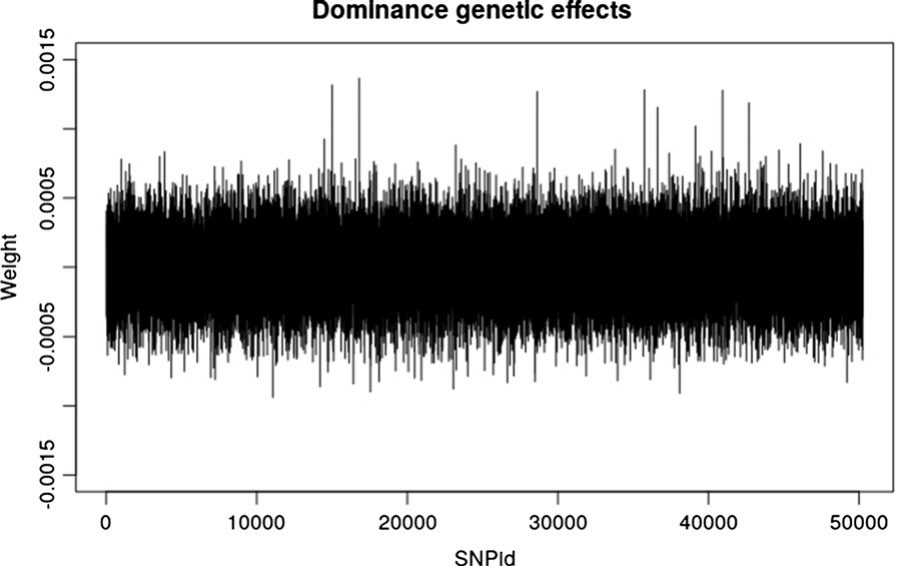
Model-averaged weight plots for the dominance genetic effects for the best model (one-neuron, one-layer and linear activation function) on the Cleveland pig dataset

The model-averaged weights were extracted from the best model. Based on these, additive and dominance genetic effects were calculated and plotted (Figs. 4, 5), which shows that six SNPs have quite high dominance effects. The same SNPs were found to be important in an earlier study [34].

## Discussion

Alternative designs to fully-connected feed-forward NN differ in the way neurons are arranged and activated, and the architecture needs to be tailored to specific applications. Shallow NN have few layers and neurons, whereas deep NN consist of many layers, often with a large number of neurons, connected in various patterns. A one-layer and one-neuron NN with a linear activation function is equivalent to a standard multiple regression model. Many different architectures have been developed for deep-learning, the most important being convolutional neural networks (CNN), which are widely used for modelling images [35], and recurrent neural networks (RNN) for sequential data [36]. Restricted Boltzmann machines and autoencoders have been developed for unsupervised learning [37]. The number of software packages for deep-learning and computational sophistication have increased in recent years. However, application of NN in genetics is still relatively scarce, but tends to increase [8].

Here, we showed that regularization of genome-wide data can be obtained by a combination of weight decay and dropout in NN, and that this provides an efficient method, which can be used both for GWP and GWAS. Extensions to more complex NN resulted in overfitting and worse prediction accuracy on both simulated and real data. Hence, one can argue that it is more important to focus on efficient regularization and sparsity than on modelling of complex structures when genomic data consists of SNPs. Glória et al. [19] also found that more complex NN designs reduced the predictive accuracy compared to a simple one-layer one-node net when evaluated on simulated genotype/phenotype data.

A recommended strategy for optimization of the NN structure and the associated weight decays is to start with a simple model with one layer and one node, and monitor the MSE over a range of weight decays. Then, one can increase the complexity of the NN and evaluate if the test MSE decreases. Of course, cross-validation or some other form of test data is needed to obtain the minimum test MSE. The dropout probability can be set to a value between 0 and 1, but it turned out that the best result in terms of minimum test MSE was always obtained when setting this parameter to 0.5. Although, one should be aware that one of the dimension reduction properties of a NN structure is that once a variable from a layer is dropped, all the terms that are above it in the network also disappear [29]. Initially, it is important to evaluate values of the weight decay and the dropout using a broad range of parameter values and then consecutively shorten down the interval.

A positive outcome of our results is that the weights can be interpreted as regression coefficients and therefore will be useful for the identification of important SNPs which is the main goal of GWAS. The one-hot encoding means that both additive and dominance effects can be detected as illustrated by the figures of both datasets. It is much more difficult to interpret weights for complex NN that include non-linear activation functions, pooling and feedback. Hence, the usefulness of deep-learning for GWAS is limited, although some techniques exist for variable importance analysis e.g. [19, 38].

Although SNPs are positioned on chromosomes, it is often sufficient to assume that the SNPs behave as independent data units due to recombination. However, it should be pointed out that the structures of SNP chip data and DNA sequence data differ. Some recent studies have tried to account for the structure at the DNA level in various prediction settings. Alipanahi et al. [39] introduced the DeepBind model based on deep CNN for the detection of protein binding sites in DNA sequences. The DeepBind model was shown to outperform other methods, to recover known and novel sequence motifs, and quantify the result of sequence alterations and detect functional single-nucleotide variants (SNVs). Quang and Xie [40] proposed DanQ, a novel hybrid convolutional and bi-directional long short-term memory recurrent (LSTM) neural network framework for predicting non-coding function de novo from DNA sequences. The idea behind DanQ is that the convolution layer captures regulatory motifs, while the recurrent layer takes care of long-term dependencies between the motifs. DanQ was shown to have outstanding prediction properties. It is likely that both CNN and RNN will become more important in the near future for GWP as sequence data becomes more abundant, but the computational demands for whole-genome analyses of large samples of individuals will be huge.

In future studies, it would be interesting to combine the ABNN with convolutional and recurrent structures in order to incorporate possible LD. Another option that would be worth testing would be to replace weight decay (i.e. ridge regularization) with the ${\ell}_{1}$ penalty (i.e. lasso regularization) of the weight parameters. Further studies on other datasets are also needed before general conclusions can be drawn.

## Conclusions

This study shows how the drop-out technique can be applied to neural networks and result in an approximate Bayesian (ABNN) model that provides a computationally efficient approach with good prediction performance. ABNN is suitable for prediction of unknown phenotypes using large-scale genome-wide SNP data, and as a tool for the detection of the SNPs that contribute information to the prediction when simple linear NN are favored. Our results show that, compared with the GBLUP and BLASSO methods on simulated data and real pig data, ABNN has lower prediction error and that a simple one-neuron one-layer network is preferred over deeper and more complex structures.
